# Supplementary material for: Analysis of PPARγ Signaling Activity in Psoriasis
Source: Int J Mol Sci. 2021 Aug 10;22(16):8603. doi: 10.3390/ijms22168603 (PMC8395241; doi:10.3390/ijms22168603)
Supplement: Supplementary file 1 [file ijms-22-08603-s001.zip › Supplemental materials_Analysis of PPARg signaling activity in psoriasis/Pathway models/Models images and html files/Model of PPARG signaling in psoriasis/11410.html]

TGFA


# Protein TGFA

|  |  |
| --- | --- |
| URN | urn:agi-llid:7039 |
| Total Entities | 0 |
| Connectivity | 3423 |
| Name | TGFA |
| Description | transforming growth factor alpha |
| Notes | This gene encodes a growth factor that is a ligand for the epidermal growth factor receptor, which activates a signaling pathway for cell proliferation, differentiation and development. This protein may act as either a transmembrane-bound ligand or a soluble ligand. This gene has been associated with many types of cancers, and it may also be involved in some cases of cleft lip/palate. Alternatively spliced transcript variants encoding different isoforms have been found for this gene. [provided by RefSeq, Sep 2011] |
| Primary Cell Localization | Extracellular |
| Class | Ligand |

---

|  |  |
| --- | --- |
| Pathway | Prostaglandin E2 and Histamine in Gastric Acid Secretion |
|  | Gastric and Pancreatic Lipase Function |
|  | G0/G1 Phase Transition |
|  | Proteins Involved in Arterial Hypertension |
|  | Proteins Involved in Pulmonary Hypertension |
|  | Proteins Involved in Myocardial Ischemia |
|  | Proteins Involved in Psoriasis |
|  | Hepatic Stellate Cells in non-Alcoholic Fatty Liver Disease |
|  | Proteins Involved in Prolactinoma |
|  | Cetuximab Resistance in Colorectal Cancer |
|  | Metastatic Colorectal Cancer |
|  | Proteins Involved in Ulcerative Colitis |
|  | Vascularization in Hepatocellular Carcinoma |
|  | Proteins Involved in Hepatocellular Carcinoma |
|  | Hepatocellular Carcinoma |
|  | Growth Factor Signaling in Hepatocellular Carcinoma |
|  | Proteins Involved in Pancreatic Cancer |
|  | Pancreatic Ductal Carcinoma |
|  | Growth Factor Signaling in Pancreatic Neoplasms |
|  | Pancreatic Neuroendocrine Tumors |
|  | Goblet-Cell Related Mucus Secretion in Asthma |
|  | Mucin Production in Goblet Airway Epithelial Cells |
|  | Proteins Involved in Polycystic Ovary Syndrome |
|  | Glioblastoma, Primary |
|  | Glioblastoma, Secondary |
|  | Astrocytoma |
|  | Glioma Invasion Signaling |
|  | Proteins Involved in Glioma |
|  | Proteins Involved in Neuroblastoma |
|  | Proteins Involved in Breast Cancer Related to ERBB2/VEGFR/Akt Signaling Pathway |
|  | ERBB/VEGFR/Akt Signaling in Breast Cancer |
|  | ESR1/ERBB Positive Luminal Breast Cancer |
|  | Breast Cancer |
|  | Endometrial Cancer |
|  | Endometrioid Endometrial Cancer |
|  | Proteins Involved in Endometrial Cancer |
|  | Clear Cell Endometrial Cancer and Papillary Serous Endometrial Cancer |
|  | Proteins with Altered Expression in Endometrial Cancer |
|  | Endometrioid Ovarian Carcinoma |
|  | Proteins Involved in Endometrioid Ovarian Carcinoma |
|  | Proteins Involved in Mucinous Ovarian Carcinoma |
|  | High-Grade Serous Ovarian Carcinoma |
|  | Low-Grade Serous Ovarian Carcinoma |
|  | Proteins Involved in Low-Grade Serous Ovarian Carcinoma |
|  | Ovarian Cancer |
|  | Mucinous Ovarian Carcinoma |
|  | Proteins Involved in Cholesteatoma |
|  | Subepithelial Fibroblasts in Middle Ear Cholesteatoma |
|  | Cholesteatoma |
|  | Proteins Involved in Pulmonary Emphysema |
|  | Mucin Hyperproduction in Goblet and Mucous Cells |
|  | Proteins with Altered Expression in Cystic Fibrosis |
|  | Proteins Involved in Polycystic Kidney Disease |
|  | EGF -> CTNN Expression Targets |
|  | EGF -> AP-1/ATF Expression Targets |
|  | EGF -> CREB/CREBBP/ELK/SRF/MYC Expression Targets |
|  | EGF -> MEF/MYOD/NFATC Expression Targets |
|  | EGF -> STAT Expression Targets |
|  | EGFR -> NCOR2 Signaling |
|  | EGFR/ERBB3 -> MEF/MYOD/NFATC/MYOG Signaling |
|  | EGFR -> CTNND Signaling |
|  | EGFR/ERBB2 -> TP53 Signaling |
|  | EGFR/ERBB2 -> CTNNB Signaling |
|  | EGFR -> SMAD1 Signaling |
|  | EGFR/ERBB2 -> HIF1A Signaling |
|  | EGFR -> ZNF259 Signaling |
|  | EGFR -> AP-1/ATF2 Signaling |
|  | EGFR -> AP-1/CREB/ELK/SRF/MYC Signaling |
|  | EGFR/ERBB -> STAT Signaling |
|  | EGFR Transactivation in Cancer and Non-Cancer Cells |
|  | Receptors and Adaptor Proteins Activated in Cancer |
|  | Proteins with Altered Expression in Cancer-Associated Sustaining of Proliferative Signaling |
|  | G0/G1 Cell Cycle Phase Transition Activation in Cancer |
|  | IGF1 -> MEF/MYOD/MYOG Expression Targets |
|  | IGF1 -> STAT Expression Targets |
|  | IGF1 -> ELK/SRF/HIF1A/MYC/SREBF Expression Targets |
|  | Insulin -> STAT Expression Targets |
|  | Insulin -> CEBPA/CTNNB/FOXA/FOXO Expression Targets |
|  | Insulin -> MEF/MYOD Expression Targets |
|  | Insulin -> ELK/SRF/HIF1A/MYC/SREBF Expression Targets |
|  | EGFR -> Expression Targets in Skin |
|  | Leptin -> STAT Expression Targets |
|  | Leptin -> ELK/SRF Expression Targets |
|  | NRG1 -> EP300/ETS/ETV/SP1 Expression Targets |
|  | NRG1 -> CREB/CREBBP/ELK/SRF/MYC Expression Targets |
|  | NRG1/Catenin Expression Targets |
|  | NRG1 -> STAT Expression Targets |
|  | NRG1 -> AP-1/ATF Expression Targets |
|  | NTF3 Expression Targets |
|  | PRL/GHR -> NF/kB/ELK/SRF/MYC Expression Targets |
|  | Elevated Receptors -> Expression Targets in Breast |
|  | Estrogens/ESR1 Genomic Canonical Signaling |
|  | EGFR Signaling |
|  | TGFA -> STAT Expression Targets |
|  | TGFA -> FOXO3A Expression Targets |
|  | TGFA -> CTNNB/CTNND Expression Targets |
|  | TGFA -> TP53 Expression Targets |
|  | TGFA -> HIF1A Expression Targets |
|  | TGFA -> MEF/MYOD/NFATC Expression Targets |
|  | TGFA/AP-1/ATF Expression Targets |
|  | TGFA -> CREB/CREBBP/ELK-SRF/MYC Expression Targets |
|  | IL1B Expression Targets |
|  | IL1A Expression Targets |
|  | Dioxin Induced Chloracne (Hypothesis) |
|  | prarg negative regulators, ps-positive |
|  | PPARG negative regulators |
|  | pparg expr target\_regulators |
|  | Neighbors of disease exacerbation |
|  | Neighbors of tumor progression |
|  | Neighbors of cancer progression |
|  | Neighbors of overall survival |
|  | Neighbors of disease severity |
|  | Neighbors of tumor size |
|  | Neighbors of lesion size |
|  | Neighbors of survival rate |
|  | Neighbors of infarct size |
|  | Neighbors of survival time |
|  | Neighbors of functional recovery |
|  | Neighbors of disease-free survival |
|  | Neighbors of long-term survival |
|  | Neighbors of body weight |
|  | Neighbors of body weight gain |
|  | Neighbors of microvessel density |
|  | Neighbors of tumor promotion |
|  | Neighbors of radioresistance |
|  | Neighbors of lesion area |
|  | Neighbors of cell density |
|  | Neighbors of cancer-specific survival |
|  | Neighbors of progression-free survival |
|  | Neighbors of apoptosis rate |
|  | Neighbors of vascular density |
|  | Neighbors of cancer risk |
|  | Neighbors of cancer incidence |
|  | Neighbors of tumor aggressiveness |
|  | Neighbors of median survival |
|  | Neighbors of fasting plasma glucose |
|  | Neighbors of glucose level |
|  | Neighbors of proliferation index |
|  | Neighbors of pregnancy outcome |
|  | Neighbors of functional status |
|  | Neighbors of skin thickness |
|  | Neighbors of fibroblast phenotype |
|  | Neighbors of disease exacerbation |
|  | Neighbors of tumor progression |
|  | Neighbors of cancer progression |
|  | Neighbors of overall survival |
|  | Neighbors of disease severity |
|  | Neighbors of tumor size |
|  | Neighbors of lesion size |
|  | Neighbors of survival rate |
|  | Neighbors of infarct size |
|  | Neighbors of survival time |
|  | Neighbors of functional recovery |
|  | Neighbors of disease-free survival |
|  | Neighbors of long-term survival |
|  | Neighbors of body weight |
|  | Neighbors of body weight gain |
|  | Neighbors of microvessel density |
|  | Neighbors of tumor promotion |
|  | Neighbors of radioresistance |
|  | Neighbors of lesion area |
|  | Neighbors of cell density |
|  | Neighbors of cancer-specific survival |
|  | Neighbors of progression-free survival |
|  | Neighbors of apoptosis rate |
|  | Neighbors of vascular density |
|  | Neighbors of cancer risk |
|  | Neighbors of cancer incidence |
|  | Neighbors of tumor aggressiveness |
|  | Neighbors of median survival |
|  | Neighbors of fasting plasma glucose |
|  | Neighbors of glucose level |
|  | Neighbors of proliferation index |
|  | Neighbors of pregnancy outcome |
|  | Neighbors of functional status |
|  | Neighbors of skin thickness |
|  | Neighbors of fibroblast phenotype |
|  | Neighbors of cell development |
|  | Neighbors of immune response |
|  | Neighbors of cell population |
|  | Neighbors of T-cell development |
|  | Neighbors of inflammatory response |
|  | Neighbors of wound healing |
|  | Neighbors of angiogenesis |
|  | Neighbors of innate immune response |
|  | Neighbors of cell function |
|  | Neighbors of cell formation |
|  | Neighbors of osteoclast development |
|  | Neighbors of epithelial to mesenchymal transition |
|  | Neighbors of immunity |
|  | Neighbors of cell proliferative response |
|  | Neighbors of chemotaxis |
|  | Neighbors of cell survival |
|  | Neighbors of osteoclast differentiation |
|  | Neighbors of cell phenotype |
|  | Neighbors of tissue remodeling |
|  | Neighbors of cell adhesion |
|  | Neighbors of endothelial cell proliferation |
|  | Neighbors of fibrogenesis |
|  | Neighbors of tumor growth |
|  | Neighbors of tissue repair |
|  | Neighbors of cell count |
|  | Neighbors of cell interaction |
|  | Neighbors of SMC proliferation |
|  | Neighbors of lumen formation |
|  | Neighbors of endothelial cell migration |
|  | Neighbors of cell damage |
|  | Neighbors of pregnancy |
|  | Neighbors of aging |
|  | Neighbors of cell motility |
|  | Neighbors of bone resorption |
|  | Neighbors of fibroblast proliferation |
|  | Neighbors of adipocyte differentiation |
|  | Neighbors of microglial activation |
|  | Neighbors of regeneration |
|  | Neighbors of cell migration |
|  | Neighbors of cancer growth |
|  | Neighbors of stem cell proliferation |
|  | Neighbors of smooth muscle cell migration |
|  | Neighbors of hepatic regeneration |
|  | Neighbors of osteoclast formation |
|  | Neighbors of cell invasion |
|  | Neighbors of heart function |
|  | Neighbors of hemopoiesis |
|  | Neighbors of bone remodeling |
|  | Neighbors of ossification |
|  | Neighbors of lung development |
|  | Neighbors of autophagy |
|  | Neighbors of transcription activation |
|  | Neighbors of senescence |
|  | Neighbors of hepatocyte proliferation |
|  | Neighbors of cell transdifferentiation |
|  | Neighbors of neuroprotection |
|  | Neighbors of stem cell differentiation |
|  | Neighbors of neuronal death |
|  | Neighbors of cell growth |
|  | Neighbors of ROS generation |
|  | Neighbors of epithelial cell proliferation |
|  | Neighbors of first trimester pregnancy |
|  | Neighbors of cancer cell growth |
|  | Neighbors of cellular immune response |
|  | Neighbors of colony formation |
|  | Neighbors of cell differentiation |
|  | Neighbors of keratinocyte proliferation |
|  | Neighbors of stem cell migration |
|  | Neighbors of life span |
|  | Neighbors of psoriasis |
|  | Neighbors of inflammatory disease |
|  | Neighbors of rheumatoid arthritis |
|  | Neighbors of atherosclerosis |
|  | Neighbors of fibrosis |
|  | Neighbors of colitis |
|  | Neighbors of inflammatory bowel disease |
|  | Neighbors of injury |
|  | Neighbors of vascular remodeling |
|  | Neighbors of metastasis |
|  | Neighbors of carcinogenesis |
|  | Neighbors of multiple sclerosis |
|  | Neighbors of inflammation |
|  | Neighbors of tumor microenvironment |
|  | Neighbors of pathological angiogenesis |
|  | Neighbors of vascular disease |
|  | Neighbors of asthma |
|  | Neighbors of pneumonia |
|  | Neighbors of hyperplasia |
|  | Neighbors of osteoarthritis |
|  | Neighbors of liver fibrosis |
|  | Neighbors of myocardial infarction |
|  | Neighbors of reperfusion injury |
|  | Neighbors of airway inflammation |
|  | Neighbors of renal injury |
|  | Neighbors of liver injury |
|  | Neighbors of death |
|  | Neighbors of encephalomyelitis |
|  | Neighbors of ischemic stroke |
|  | Neighbors of dermatitis |
|  | Neighbors of diabetic nephropathy |
|  | Neighbors of lung injury |
|  | Neighbors of hepatocellular carcinoma |
|  | Neighbors of acute lung injury |
|  | Neighbors of gastric cancer |
|  | Neighbors of breast cancer |
|  | Neighbors of ischemia |
|  | Neighbors of systemic lupus erythematosus |
|  | Neighbors of cartilage degeneration |
|  | Neighbors of interstitial fibrosis |
|  | Neighbors of kidney disease |
|  | Neighbors of lung disease |
|  | Neighbors of pulmonary fibrosis |
|  | Neighbors of stroke |
|  | Neighbors of cancer |
|  | Neighbors of cerebral ischemia |
|  | Neighbors of type 2 diabetes |
|  | Neighbors of neoplasm |
|  | Neighbors of atrophy |
|  | Neighbors of respiratory hypersensitivity |
|  | Neighbors of hypertrophy |
|  | Neighbors of osteolysis |
|  | Neighbors of ovarian cancer |
|  | Neighbors of pancreatic cancer |
|  | Neighbors of hypertension |
|  | Neighbors of epithelial cell |
|  | Neighbors of keratinocyte |
|  | Neighbors of monocyte |
|  | Neighbors of endometrial stromal cell |
|  | Neighbors of alveolar macrophage |
|  | Neighbors of macrophage |
|  | Neighbors of fibroblast |
|  | Neighbors of airway epithelial cell |
|  | Neighbors of PBMC |
|  | Neighbors of stromal cell |
|  | Neighbors of osteoblast |
|  | Neighbors of pneumocyte |
|  | Neighbors of microglia |
|  | Neighbors of intestine epithelium cell |
|  | Neighbors of mononuclear cell |
|  | Neighbors of hepatocyte |
|  | Neighbors of endothelial cell |
|  | Neighbors of Kuppfer cell |
|  | Neighbors of inflammatory cell |
|  | Neighbors of neutrophil |
|  | Neighbors of astrocyte |
|  | Neighbors of lung fibroblast |
|  | Neighbors of mesothelial cell |
|  | Neighbors of granulocyte |
|  | Neighbors of cancer-associated fibroblast |
|  | Neighbors of myeloid cell |
|  | Neighbors of corneal epithelial cell |
|  | Neighbors of cytotrophoblast |
|  | Neighbors of stem cell |
|  | Neighbors of granulosa cell |
|  | Neighbors of leukocyte |
|  | Neighbors of chondrocyte |
|  | Neighbors of mesenchymal cell |
|  | Neighbors of T-cell |
|  | Neighbors of myofibroblast |
|  | Neighbors of mast cell |
|  | Neighbors of neuroglia |
|  | Neighbors of mammary epithelial cell |
|  | Neighbors of epidermis cell |
|  | Neighbors of liver |
|  | Neighbors of kidney |
|  | Neighbors of skin |
|  | Neighbors of heart |
|  | Neighbors of gastrointestinal tract |
|  | Neighbors of esophagus |
|  | Neighbors of stomach |
|  | Neighbors of prostate |
|  | Neighbors of villus |
|  | Neighbors of glands |
|  | Neighbors of eye |
|  | Neighbors of brain blood vessel |
|  | Neighbors of hypothalamus |
|  | Neighbors of choroid plexus |
|  | Neighbors of NF-kB family |
|  | Neighbors of NFKBI |
|  | Neighbors of PKC |
|  | Neighbors of PI3K |
|  | Neighbors of transforming growth factor |
|  | Neighbors of cytokine |
|  | Neighbors of interferon |
|  | Neighbors of interleukin |
|  | Neighbors of JAK |
|  | Neighbors of Ras GTPase |
|  | Neighbors of growth factor |
|  | Neighbors of PKA |
|  | Neighbors of LDL |
|  | Neighbors of chorionic gonadotropin |
|  | Neighbors of NAD(P)H oxidase |
|  | Neighbors of estrogen receptor |
|  | Neighbors of HIF-1 |
|  | Neighbors of AHR subfamily |
|  | Neighbors of FSH |
|  | Neighbors of TLR4/Myd88 |
|  | Model of PPARG signaling in psoriasis |
|  | 2\_Pathogens stimulate mucins expression in the middle ear |
|  | 3\_1\_Mucus accumulation in asthma |
|  | 4\_Mucin hyperproduction in goblet and mucous cells in COPD |

---

|  |  |
| --- | --- |
| Group | Chemokines, Growth Factors and Receptors Involved in Cancer Cells Chemotaxis |

---

|  |  |
| --- | --- |
| MedScan ID | 7039 |

---

|  |  |
| --- | --- |
| LocusLink ID | 7039 |
|  | 21802 |
|  | 24827 |

---

|  |  |
| --- | --- |
| Alias | OTTHUMP00000203352 |
|  | TGFAA |
|  | OTTHUMP00000203300 |
|  | 1MOX\_Ds |
|  | TGFA |
|  | LOC150914 |
|  | protransforming growth factor alpha |
|  | EGF-like TGF |
|  | RATTGFAA |
|  | TGF-alpha |
|  | OTTHUMP00000203345 |
|  | LOC130089 |
|  | waved 1 |
|  | Transforming Growth Factor-alphahuman |
|  | TGF A |
|  | 1MOX\_D |
|  | wa I |
|  | type alpha transforming growth factor |
|  | OTTHUMP00000203301 |
|  | ETGF |
|  | TGFA\_HUMAN |
|  | Transforming growth factor alpha |
|  | tfga |
|  | wa1 |
|  | wa-1 |
|  | transforming growth factor, alpha |
|  | transforming growth factor-alpha |
|  | waved I |

---

|  |  |
| --- | --- |
| GO ID | 0005154 |
|  | 0008083 |
|  | 0048208 |
|  | 0006888 |
|  | 0000165 |
|  | 0000187 |
|  | 0007173 |
|  | 0035556 |
|  | 0061024 |
|  | 0042059 |
|  | 0051781 |
|  | 0008284 |
|  | 0045741 |
|  | 0050679 |
|  | 0045840 |
|  | 0051897 |
|  | 0006357 |
|  | 0007165 |
|  | 0012507 |
|  | 0000139 |
|  | 0016323 |
|  | 0009986 |
|  | 0030665 |
|  | 0031410 |
|  | 0005789 |
|  | 0033116 |
|  | 0005576 |
|  | 0005615 |
|  | 0016021 |
|  | 0048471 |
|  | 0005886 |
|  | 0001525 |
|  | 0072574 |
|  | 0060749 |
|  | 0043066 |
|  | 0048523 |
|  | 0050731 |
|  | 0016020 |
|  | 0005634 |
|  | 0042493 |
|  | 0042060 |
|  | 0005887 |
|  | 0008283 |
|  | 0001948 |
|  | 0004708 |
|  | 0051480 |
|  | 0043524 |
|  | 0006916 |
|  | 0004871 |
|  | 0043526 |
|  | 0001934 |

---

|  |  |
| --- | --- |
| KEGG ID | hsa:7039 |
|  | mmu:21802 |
|  | rno:24827 |

---

|  |  |
| --- | --- |
| Organism | Homo sapiens {Organism urn:agi-taxid:9606} |
|  | Mus musculus {Organism urn:agi-taxid:10090} |
|  | Rattus norvegicus {Organism urn:agi-taxid:10116} |
|  | Homo sapiens |
|  | Mus musculus |
|  | Rattus norvegicus |

---

|  |  |
| --- | --- |
| Mouse chromosome position | 6 37.62 cM |
|  | 6 35.8 cM |

---

|  |  |
| --- | --- |
| OMIM ID | 190170 |

---

|  |  |
| --- | --- |
| Rat chromosome position | 4q34 |

---

|  |  |
| --- | --- |
| Hugo ID | 11765 |
|  | HGNC:11765 |

---

|  |  |
| --- | --- |
| Human chromosome position | 2p13.3 |
|  | 2p13 |

---

|  |  |
| --- | --- |
| Swiss-Prot Accession | P01135 |
|  | F8VNR3 |
|  | E7EPT6 |
|  | P01135.1 |
|  | P48030 |
|  | Q545E4 |
|  | P48030.1 |
|  | P01134 |
|  | U5LKN7 |
|  | P01134.2 |
|  | A8K286 |
|  | Q15577 |
|  | Q53SK7 |
|  | Q9BS56 |
|  | Q9UEI3 |
|  | Q9UKM1 |
|  | Q9UKM2 |
|  | Q9UKM3 |
|  | Q63749 |
|  | Q15578 |
|  | Q6QBS1 |
|  | Q9UE87 |
|  | Q9UIS1 |
|  | Q9UIS2 |
|  | Q9UIS3 |
|  | Q9UQ91 |
|  | Q6LC37 |

---

|  |  |
| --- | --- |
| PIR ID | JN0876 |
|  | S27195 |
|  | A93356 |
|  | I57497 |

---

|  |  |
| --- | --- |
| GenBank ID | NC\_000002 |
|  | NM\_001099691 |
|  | NP\_001093161 |
|  | NM\_003236 |
|  | NP\_003227 |
|  | NM\_001308159 |
|  | NP\_001295088 |
|  | NM\_001308158 |
|  | NP\_001295087 |
|  | NG\_029975 |
|  | AC005234 |
|  | AAY14793 |
|  | AC017084 |
|  | AAY14705 |
|  | AH003012 |
|  | AAA61158 |
|  | AH007317 |
|  | AAD12238 |
|  | AAD12239 |
|  | AH008373 |
|  | AAF06728 |
|  | AAF06729 |
|  | AAF06730 |
|  | AH008439 |
|  | AAF13491 |
|  | AH013033 |
|  | AAP97822 |
|  | CH471053 |
|  | EAW99810 |
|  | EAW99811 |
|  | EAW99812 |
|  | EAW99813 |
|  | EAW99814 |
|  | M22440 |
|  | AAA52530 |
|  | M96868 |
|  | AAA79958 |
|  | X70341 |
|  | AF149096 |
|  | AAF05089 |
|  | AF149097 |
|  | AAF05090 |
|  | AF149098 |
|  | AAF05091 |
|  | AK290151 |
|  | BAF82840 |
|  | AK309066 |
|  | AK309220 |
|  | AK312899 |
|  | AY548763 |
|  | AAS83396 |
|  | BC005308 |
|  | AAH05308 |
|  | BI760767 |
|  | BM453873 |
|  | BT006833 |
|  | AAP35479 |
|  | CD369689 |
|  | CN274505 |
|  | DA216480 |
|  | DA229819 |
|  | DA373586 |
|  | DA462271 |
|  | K03222 |
|  | AAA61159 |
|  | M31172 |
|  | AAA61157 |
|  | X70340 |
|  | CAA49806 |
|  | P01135 |
|  | NC\_000072 |
|  | NM\_031199 |
|  | NP\_112476 |
|  | AC159549 |
|  | CH466523 |
|  | EDK99158 |
|  | EDK99159 |
|  | U64873 |
|  | AAB50553 |
|  | U89794 |
|  | AAC53174 |
|  | AK010785 |
|  | BAB27181 |
|  | AK162680 |
|  | BAE37019 |
|  | AK165017 |
|  | AK166321 |
|  | BAE38704 |
|  | BC132185 |
|  | AAI32186 |
|  | BC132211 |
|  | AAI32212 |
|  | BE848968 |
|  | M92420 |
|  | AAA40481 |
|  | U65016 |
|  | AAB50554 |
|  | P48030 |
|  | NC\_005103 |
|  | XM\_006236822 |
|  | XP\_006236884 |
|  | NM\_012671 |
|  | NP\_036803 |
|  | AC\_000072 |
|  | AABR07061351 |
|  | AABR07061352 |
|  | AABR07061353 |
|  | AAHX01031356 |
|  | AAHX01031357 |
|  | AAHX01031358 |
|  | AAHX01031359 |
|  | CH473957 |
|  | EDL91222 |
|  | EDL91223 |
|  | EDL91224 |
|  | M31075 |
|  | AAA42234 |
|  | KF366251 |
|  | AGX26150 |
|  | M31076 |
|  | AAA42233 |
|  | X02004 |
|  | CAA26036 |
|  | X61485 |
|  | X61486 |
|  | X61487 |
|  | P01134 |
|  | XM\_005264522 |
|  | XP\_005264579 |
|  | XM\_011533076 |
|  | XP\_011531378 |
|  | NC\_018913 |
|  | AF075583 |
|  | AF075584 |
|  | AF075585 |
|  | AF075586 |
|  | AF123238 |
|  | AF123239 |
|  | AF123240 |
|  | AF123241 |
|  | AF123242 |
|  | AF123243 |
|  | AF149099 |
|  | AF149100 |
|  | AMYH02004039 |
|  | AMYH02004040 |
|  | AY325885 |
|  | AY325886 |
|  | AY326405 |
|  | AY327131 |
|  | AY327132 |
|  | AY329368 |
|  | K03220 |
|  | K03221 |
|  | AC\_000028 |
|  | AAHY01055020 |
|  | AAHY01055021 |
|  | AAHY01055022 |
|  | AAHY01055023 |
|  | AAHY01055024 |
|  | XM\_008763049 |
|  | XP\_008761271 |
|  | XM\_008763050 |
|  | XP\_008761272 |
|  | AC\_000134 |
|  | ABBA01075538 |
|  | ABBA01075539 |
|  | NT\_022184 |
|  | NW\_001838769 |
|  | NW\_927719 |
|  | AC\_000045 |
|  | Q15578 |
|  | Q6QBS1 |
|  | Q9UE87 |
|  | Q9UIS1 |
|  | Q9UIS2 |
|  | Q9UIS3 |
|  | Q9UKM3 |
|  | Q9UQ91 |
|  | NT\_039353 |
|  | NW\_001030811 |
|  | Q545E4 |
|  | Q6LC37 |
|  | NW\_047694 |
|  | NW\_001084830 |

---

|  |  |
| --- | --- |
| Swiss-Prot ID | TGFA\_HUMAN |
|  | TGFA\_MOUSE |
|  | TGFA\_RAT |
|  | Q545E4\_MOUSE |
|  | A8K286\_HUMAN |
|  | Q9BS56\_HUMAN |

---

|  |  |
| --- | --- |
| Cell Localization | Secreted |
|  | Extracellular space |
|  | Cell membrane |
|  | Secreted, extracellular space |
|  | Extracellular |

---

|  |  |
| --- | --- |
| Ensembl ID | ENSG00000163235 |
|  | ENSP00000404099.2 |
|  | ENST00000418333.6 |
|  | ENSP00000404131.1 |
|  | ENST00000444975.5 |
|  | ENSP00000414127.1 |
|  | ENST00000450929.5 |
|  | ENSP00000295400.6 |
|  | ENST00000295400.11 |
|  | ENSMUSG00000029999 |
|  | ENSMUSP00000032066.9 |
|  | ENSMUST00000032066.12 |
|  | ENSRNOG00000016182 |
|  | ENSRNOP00000054248.2 |
|  | ENSRNOT00000057441.4 |
|  | ENSP00000404099 |
|  | ENST00000418333 |
|  | ENSP00000295400 |
|  | ENST00000295400 |
|  | ENSP00000404131 |
|  | ENST00000444975 |
|  | ENSP00000414127 |
|  | ENST00000450929 |
|  | ENSMUSP00000032066 |
|  | ENSMUST00000032066 |
|  | ENSRNOP00000054248 |
|  | ENSRNOT00000057441 |

---

|  |  |
| --- | --- |
| MGI ID | MGI:98724 |
|  | 98724 |

---

|  |  |
| --- | --- |
| RGD ID | 3849 |

---

|  |  |
| --- | --- |
| Unigene ID | Hs.170009 |
|  | Hs.628298 |
|  | Mm.137222 |
|  | Rn.9952 |

---

|  |  |
| --- | --- |
| Homologene ID | 2431 |

---

|  |  |
| --- | --- |
| Shape | Rhomb |

---

|  |  |
| --- | --- |
| IPI ID | IPI00137996 |
|  | IPI00465177 |
|  | IPI00856067 |
|  | IPI00872280 |
|  | IPI00926333 |
|  | IPI00954043 |
|  | IPI00779017 |
|  | IPI00462181 |
|  | IPI00327408 |

---
